# Obstetric care for environmental migrants

**DOI:** 10.1007/s11845-023-03481-9

**Published:** 2023-09-16

**Authors:** Sadhbh A. Lee, Gillian A. Corbett, Fionnuala M. McAuliffe

**Affiliations:** 1 National Maternity Hospital, Holles St., Dublin 2, Ireland; 2 UCD Perinatal Research Centre, School of Medicine, University College Dublin, National Maternity Hospital, Dublin, Ireland

**Keywords:** Climate change, Environmental migration, Migration, Natural disasters, Obstetrics and gynaecology, Pregnancy

## Abstract

**Background:**

Migration due to environmental factors is an international crisis affecting many nations globally. Pregnant people are a vulnerable subgroup of migrants.

**Aim:**

This article explores the potential effects of environmental migration on pregnancy and aims to draw attention to this rising concern.

**Methods:**

Based on the study aim, a semi-structured literature review was performed. The following databases were searched: MEDLine (PubMed) and Google Scholar. The search was originally conducted on 31st January 2021 and repeated on 22nd September 2022.

**Results:**

Pregnant migrants are at increased risk of mental health disorders, congenital anomalies, preterm birth, and maternal mortality. Pregnancies exposed to natural disasters are at risk of low birth weight, preterm birth, hypertensive disorders, gestational diabetes, and mental health morbidity. Along with the health risks, there are additional complex social factors affecting healthcare engagement in this population.

**Conclusion:**

Maternity healthcare providers are likely to provide care for environmental migrants over the coming years. Environmental disasters and migration as individual factors have complex effects on perinatal health, and environmental migrants may be at risk of specific perinatal complications. Obstetricians and maternity healthcare workers should be aware of these challenges and appreciate the individualised and specialised care that these patients require.

## Introduction

Environmental migration is emerging as a concerning issue for the global population. Environmental disasters have always been a major cause of human migration globally. In today’s world, with climate change predicted to cause increasingly frequent and severe natural disasters, along with other environmental factors such as rising temperatures and air pollution, the number of people displaced due to environment-related reasons is predicted to rise over the coming years [1]. Analysts have estimated that there will be up to two hundred million environmental migrants by 2050 [2]. Environmental migrants are defined by the International Organization of Migration as “persons or groups of persons who, predominantly for reasons of sudden or progressive changes in the environment that adversely affect their lives or living conditions, are obliged to leave their habitual homes, or choose to do so, either temporarily or permanently, and who move within their own country or abroad.” [3]. In 2019, the Internal Displacement Monitoring Centre recorded twenty-eight million internal displacements (those displaced within their own country) across one hundred and forty-eight countries and territories, with 17·2 million of these related to natural disasters [4]. The regions most affected were East Asia and Pacific, South Asia, and sub-Saharan Africa, with the Philippines, China, India, USA, and Indonesia ranking as the five countries most affected. However, between increasingly frequent storms, flooding, and wildfires, there are very few countries worldwide that are not vulnerable to natural disasters.

The majority of migration in the context of environmental change happens domestically, but people are also forced to migrate across borders. Global data on this is lacking, as is data on migration due to slow-onset climate processes—environmental changes that take place over long time periods of decades, such as air pollution or sea-level rise.

Environmental migration is an important health issue. To begin with, exposure to environmental changes affects human health directly in a multitude of ways. Short-term effects are seen in the wake of natural disasters and include death, injury, gastro-intestinal, and other infectious diseases. Long-term effects of issues like air pollution and rising temperatures include increased non-communicable diseases, spread of tropical infectious diseases to non-tropical areas, malnutrition, and mental health issues. Secondly, the context of migration can expose a person to increased health risks: death or injury from violence/travel/disaster events; infectious diseases from poor living conditions and suboptimal hygiene and nutrition; deterioration of non-communicable diseases from interruption of care; and mental health problems such as depression, post-traumatic stress disorder (PTSD), or addiction.

Of note for maternity healthcare workers, pregnant populations are particularly vulnerable to this range of health risks. The World Health Organization (WHO) 2018 report on the health of refugees and migrants in Europe recognised a marked increase in poorer pregnancy outcomes for migrant women compared with non-migrant women [5]. A systematic review by Helsehurst et al. found pregnant migrant women are at increased risk of mental health disorders, maternal mortality, preterm birth, and congenital anomalies when compared to women in the host country [6]. Migration factors contributing to adverse pregnancy outcomes include poor living conditions, poverty, unemployment, and reduced access to healthcare services.

Given that migrants have poorer pregnancy outcomes, it follows that environmental migration will have impacts on maternal morbidity. In the setting of rising environmental migration, obstetricians worldwide are likely to encounter increasing numbers of domestic and international environmental migrants over the coming years. For these two reasons, it is essential to look specifically at environmental migration in the context of pregnancy and begin to explore the unique adverse risk profile for this vulnerable cohort of patients. This will help obstetricians, midwives, and healthcare providers to recognise the need for individualised obstetric care, thus mitigating risk and optimising pregnancy outcomes for these patients. It will also allow maternity healthcare providers to take a global, holistic approach to their work, working under the framework of the United Nations’ Sustainable Development Goals (SDGs) [7]. These seventeen goals provide guidance for all countries to work to end poverty while also developing strategies that improve health, reduce inequalities, and fight climate change—exactly the approach needed to the care of environmental migrants.

## Aim

Given a lack of available data on migration due to slow-onset climate processes, this piece will focus on the pregnant population potentially affected by migration due to environmental disasters. There are four main categories of environmental disasters that cause migration, each with their own set of specific risks in pregnancy:

1. Storms and flooding
2. Geophysical—earthquakes, volcano eruptions
3. Drought
4. Wildfires

This paper aims to present the literature on the risk of these disasters on pregnancy outcomes and to investigate the potential obstetric risks faced by women migrating due to these causes. By identifying these morbidities, the obstetrician will be empowered to recognise, screen for, and mitigate these risks, thus minimising adverse outcomes for these patients.

## Methodology

Based on the study aim, a semi-structured literature review was performed. The following databases were searched: MEDLine (PubMed) and Google Scholar. The search was originally conducted on 31st January 2021 and repeated on 22nd September 2022.

## Search strategy and selection criteria

Background information relating to migration, environmental migration, climate change, and pregnancy was gathered from websites of international organisations such the United Nations, the World Health Organization, the American College of Obstetricians and Gynaecologists, the International Organization for Migration, and the United States Geological Survey. Information gathered from these sites was used to generate the following MeSH terms: “natural disaster, climate change, migration, hurricane, cyclone, flood*, earthquake, volcano, drought, wildfire, air pollution” *and* “pregnan*, perinatal, birth”. The terms were then combined using Boolean search terms (AND/OR).

For this search, inclusion and exclusion criteria were based on the study topic as follows:

**Table:** 

**Inclusion criteria**	**Exclusion criteria**
Original research articles, observational cohort studies, experimental studies, reviews	Non-human subjects
Human subjects	Effects on general, neonatal or early childhood health
Effects on maternal and perinatal health	Results not reported
Geographical diversity	

Studies that met the selection criteria were screened by title and abstract. Articles with title and abstract in keeping with the review’s aim were then moved forward for full article review. Where it was unclear from title and abstract review if the article met the inclusion criteria, it was included for full review.

All relevant studies were included regardless of language or publication status (published, unpublished, in press, or in progress). Duplicate articles were noted and removed. Reference lists for all articles yielded through database search were examined for additional articles for inclusion, including published reviews on this topic or similar topic content.

## Results

Database searches revealed 6709 articles after removal of duplicates. Ninety-six underwent abstract screening. After title and abstract review, 71 articles were considered relevant and their full manuscripts were reviewed for inclusion. Fifty-three of these studies met the criteria for inclusion. Studies are summarised in Table 1.

**Table 1:** Study characteristics and results

Natural disaster	Outcome	Year; author	Study design	Study size	Effect
Storms and flooding	Low birth weight	2009, Hilmert	Prospective study	169 pregnancies	Increased rates of LBW with first trimester flood exposure
		2010, Harville	Systematic review	49 studies	Increased rates of LBW with hurricane/flood exposure
		2010, Tong	Observational cohort study	57,007 births	Increased rates of LBW with flood exposure
		2012, Alderman	Systematic review	139 studies	Increased rates of LBW with hurricane/flood exposure
		2017, Mallett	Systematic review	141 studies	Increased rates of LBW with flood exposure
		2021, De Oliveira	Retrospective case–control study	133,513 births	Increased rates of LBW with hurricane exposure
		2021, Pan	Retrospective case–control study	433,371 births	Increased rates of LBW and SGA with hurricane exposure
		2022, Parawiya	Retrospective case–control study	647,634 births	Increased rates of LBW with mid-trimester cyclone exposure
	Preterm Birth	2010, Tong	Observational cohort study	57,007 births	Increased rates of PTB with flood exposure
		2015, Antipova	Retrospective case–control study	12,475 births	Increased rates of PTB with flood exposure
		2016, Grabich	Retrospective case–control study	342,942 births	Increased rates of PTB with high speed wind exposure
		2019, Xiao	Time-series study	Population size not specified—ED visits over a 9-year period examined (343 mean per day)	Increased ED attendances with PTB after hurricane exposure
		2020, Sun	Retrospective case–control study	19,529,748 births	Increased risk of PTB with tropical cyclone exposure
		2022, Parawiya	Retrospective case–control study	647,634 births	Increased rates of PTB with early pregnancy cyclone exposure
	Hypertensive disorders	2014, Khan	Population-based case–control study	202 pregnancies	Increased HTN disorders related to increasing water salinity from flooding
		2019, Mendez-Figueroa	Observational cohort study	29,179 births	Increased HTN disorders of pregnancy after hurricane exposure
	Malnutrition	1994, Duff	Case–control	17 mothers	Increased rates of NTDs following hurricane exposure
		2011, Goudet	Qualitative and quantitative analysis	22 mothers	Reduced rates of breast-feeding after flood exposure
	Increased rates of Caesarean section	2009, Hamilton	Descriptive report	27,848 births	6–10% increase in CS rates after hurricane exposure
		2011, Zahran	Retrospective case–control study	297,996 births	20% increase in risk of emergency CS with hurricane exposure
		2019, Mendez-Figueroa	Observational cohort study	29,179 births	5% increase in CS rates after hurricane exposure
	Increased emergency department attendances	2019, Xiao	Time-series study	Population size not specified—ED visits over a 9-year period examined (343 mean per day)	Increased ED attendances for maternal chronic disease after hurricane exposure
		2021, Xiao	Cross-sectional study	307,739 ED visits (pregnancy)	16.6% increased ED attendances for pregnancy complications in the week after hurricane exposure
		2021, Ramesh	Controlled times series study	9,047,462 ED visits (general)	24% increase in attendances for pregnancy complications after flooding
	Psychological morbidity	2009, Harville	Cohort study	292 pregnant women	Increased rates of PTSD and depression with hurricane exposure
		2012, Alderman	Systematic review	139 studies	Increased pregnancy-related anxiety with hurricane/flood exposure
		2017, Mallett	Systematic review	141 studies	Increased depression and PTSD with flood exposure
		2019, Giarrantano	Mixed-methods study	402 pregnant women	Increased depression, PTSD and anxiety with hurricane exposure
		2019, Xiao	Time-series study	Population size not specified—visits to ED examined	Increased ED attendances with mental health problems after hurricane exposure
Geophysical events	Low birth weight	2009, Tan	Observational cohort study	13,003 births	Increased LBW with earthquake exposure (*p* < 0.01)
		2010, Harville	Systematic review	49 studies	Increased LBW with earthquake exposure
		2012, Oyarzo	Retrospective observational case–control study	6162 births	Increased LBW with earthquake exposure
		2014, Fujimori	Survey-based study	8602 respondents	No increase in LBW after earthquake exposure
		2018, Sugawara	Retrospective cohort study	12,808 births	No increased in LBW after earthquake exposure
		2020, Khatri	Prospective population-based study	469 births	Increased rates of mental health disorders following earthquake exposure associated with increased LBW
	Preterm birth	2001, Glynn	Population-based observational study	40 pregnant women	Increased rates of PTB with earthquake exposure in first trimester
		2009, Tan	Observational cohort study	13,003 births	Increased rates of PTB with earthquake exposure
		2010, Harville	Systematic review	49 studies	Increased rates of PTB with earthquake exposure in first trimester
		2012, Oyarzo	Retrospective observational case–control study	6162 births	Increased rates of PTB with earthquake exposure
		2012, Torche	Retrospective observational case–control study	600,000 births	Increased rates of PTB with earthquake exposure in first trimester, more significantly for female over male births
		2016, Balsa	Retrospective observational cohort study	79,328 births	Increased rates of PTB due to third trimester exposure to air pollution from volcanic eruptions
		2018, Sugawara	Retrospective cohort study	12,808 births	No increased in PTB after earthquake exposure
		2019, Hawkins	Retrospective cohort study	2371 births	No significant difference in the rate of PTB with earthquake exposure
		2020, Lian	Retrospective cross-sectional study	73,493 births	Significant increased risk of PTB with earthquake exposure
	Hypertensive disorders	2020, Kyozuka	Survey-based study	8323 respondents	Increased risk of hypertensive disorders with earthquake exposure
	Psychological morbidity	2012, Qu	Qualitative study	311 pregnant women	High rates of depression and PTSD after earthquake exposure
		2014, Ren	Systematic review	8 articles	Depression and PTSD most commonly reported mental health disorders post-earthquake exposure
		2015, Khatri	Prospective population-based study	469 births	Higher rates of mental health disorders with earthquake exposure
Drought (dehydration/malnutrition)	Low birth weight	2015, Hanson	FIGO recommendations		Maternal micronutrient deficiency associated with increased risk of low birth weight
		2019, Hendrickson	UpToDate		Maternal macro/micronutrient deficiency associated with increased risk of low birth weight
	Preterm birth	2015, Hanson	FIGO recommendations		Maternal macro/micronutrient deficiency associated with increased risk of preterm birth
	Anaemia	2015, Hanson	FIGO recommendations		Maternal micronutrient deficiency associated with increased risk of anaemia
Wildfires	Low birth weight	2012, Holstius	Observational cohort study	886,034 births	Increased LBW with wildfire exposure, not statistically significant
		2013, O’Donnell	Population cohort study	287,688 births	Increased rates of LBW with wildfire exposure
		2019, Abdo	Observational cohort study	535,895 births	Increased rates of LBW with first trimester wildfire exposure
		2021, Amjad	Systematic review	8 studies (1,702,252 births)	Increased risk of LBW with wildfire exposure
	Preterm birth	2013, O’Donnell	Population cohort study	287,688 births	Increased rates of PTB with wildfire exposure
		2019, Abdo	Observational cohort study	535,895 births	Increased rates of PTB with wildfire exposure
		2022, Requia	Time-stratified case-crossover study	190,911 preterm births	Increased rates of PTB with wildfire exposure in the first or second trimester
		2022, Heft-Neal	Retrospective case–control study	3,002,014 births	Increased risk of PTB with wildfire smoke exposure
	Hypertensive disorders	2019, Abdo	Observational cohort study	535,895 births	Postive association between wildifre exposure and gestational hypertension
	Gestational diabetes	2019, Abdo	Observational cohort study	535,895 births	Increased rates GDM with first trimester wildfire exposure
		2020, Melody	Observational cohort study	3612 births	Increased likelihood of GDM with exposure to air pollution from coalmine fire
	Congenital abnormalities	2022, Requia	Retrospective cohort study	16,825,497 births	Increased risk of certain birth defects with wildfire exposure
		2022, Park	Retrospective cohort study	844,348 births	Increased risk of gastroschisis with wildfire exposure

Results are examined under each category of environmental disaster.

## Storms, flooding and pregnancy

A tropical cyclone is defined by the National Oceanic and Atmospheric Administration as “a rotating, organised system of clouds and thunderstorms that originates over tropical or subtropical waters” [8]. A hurricane or typhoon is a cyclone with sustained winds of over 74 mph. When these storms make landfall, they cause massive wind and flood damage, and research strongly suggests that tropical cyclones will become more intense and have worsening flooding and rainfall effects due to climate change [9, 10]. Flooding is the most common natural disaster worldwide, with floods accounting for 47% of natural disasters recorded from 1995 to 2015, affecting more than two billion people worldwide [11]. Flooding is caused by severe precipitation, rising sea levels, and extreme weather events, all of which will increase if climate change continues at its current rate. 14.7 million people were internally displaced by storms and flooding in 2019 [4].

The effects of storms and flooding may be considered together. These natural disasters lead to decreased water quantity and quality, exposure to toxicants, displacement, reduced access to health services, and food shortages [12]. Ha recently published a review on the direct and indirect effects of climate change on pregnancy, including the effects of some natural disasters [12]. Looking at a 2020 review of 19 papers on hurricane exposure and pregnancy, the author notes a number of methods used in the literature to measure exposure to hurricanes and floods: “distance from storm, property damage, residence in nationally designated disaster areas, maximum wind speed, or questionnaires with specific impact scales” [12].

### Low birth weight

Studies performed after Hurricane Andrew in Louisiana, Hurricane Katrina in New Orleans, and Hurricane Catarina in Brazil showed an association between increased exposure to hurricane and flooding events and risk of low birth weight [13–15]. A study looking at pregnancy outcomes after Hurricane Michael (2018) in Florida found a significant association with low birth weight and small-for-gestational age (SGA) [17]. A 2022 paper by Parayiwa et al. looking at birth outcomes after cyclone events in Australia found significantly higher odds of low birth weight associated with mid-trimester cyclone exposure [18]. The association has also been suggested by studies looking at flooding in the Red River area in North Dakota, where Tong et al. found a significant increase in low birth weight in the region after the 1997 flood [19]. A second study performed after flooding in the region in 2009 found an increased risk of low birth weight in women exposed to flooding in the first trimester, but not extending to those affected after 26 weeks gestation [20]. A systematic review by Harville et al. looking at the effects of disasters on perinatal health found more consistent results for negative effects of flooding on fetal growth and birth weight than on preterm birth [21].

### Preterm birth

A 2020 analysis of 20 million births in the USA found maternal exposure to tropical cyclones increased the risk of preterm birth, particularly for early preterm birth and for socially vulnerable mothers [22]. Parayiwa et al. found a significantly increased risk of preterm birth with early pregnancy cyclone exposure [18]. Rates of preterm birth rose after Hurricanes Andrew, Katrina, Charley, and Catarina and after flooding in North Dakota in 1997 [13–16, 19, 23]. Emergency department attendances for early onset delivery increased by 115% in the 2 months after Hurricane Sandy in 2012 [24]. Although there is limited data on the relationship between preterm birth and storms/flooding, it is proposed to be related to increased prenatal stress.

### Hypertensive disorders

In the aftermath of hurricanes and flooding, hypertensive disorders of pregnancy appear to be more common. After Hurricane Harvey in 2017, hypertensive disorders of pregnancy were 60% more common [25]. Although likely a multifactorial association, this may be linked to contamination of drinking water supplies. Flooding can cause sea-level rise and as a result, salinity levels in drinking water sources can become excessively high due to seawater contamination. Higher sodium levels in drinking water caused by sea-level rise were associated with higher rates of pre-eclampsia and gestational hypertension in a study conducted in Bangladesh [26].

### Malnutrition

Disruption caused to crops by flooding can lead to concerns about malnutrition. Reduced quantity and variety of food led to a reduction in milk supply in breast-feeding mothers affected by flooding in Bangladesh, while crop failure after Hurricane Gilbert in Jamaica in 1988 led to an increase in neural tube defects, considered to be related to reduced folic acid intake [13, 27].

### Increased rate of Caesarean section

Three studies demonstrated a rise in rates of Caesarean section after hurricanes: a 20% increase in Florida after Hurricane Andrew, a 5% increase in Houston after Hurricane Harvey, and a 6–10% increase in both planned and emergency sections after Hurricane Katrina [25, 28, 29]. Possible explanations are maternal stress or placental disease from hypertensive disorders and malnutrition occurring in the wake of these storms, as described above.

### Increase in emergency department attendances

Xiao et al. found significant increases in emergency department attendances for gestational hypertension, renal disease, diabetes mellitus, and abnormal glucose after Hurricane Sandy, beginning in the immediate aftermath and peaking at 7 to 8 months after the storm [24]. The authors suggest that the power outages caused by the storm led to decreased access to diabetic medication, glucose monitoring devices, and renal dialysis. A second study by Xiao et al. in 2021 also reported a 16.6% increase in emergency department attendances for pregnancy complications during power outages within 1 week of the storm [30]. Flooding after Hurricane Harvey in Texas 2017 was associated with a 24% increase in emergency department attendances for pregnancy-related issues [31].

### Psychological comorbidity

From a mental health perspective, pregnant patients exposed to hurricanes and flooding have been shown to be at increased risk of antenatal depression, post-traumatic stress disorder (PTSD), and pregnancy-related anxiety [13, 14, 32, 33]. A cohort study performed after Hurricane Katrina found postpartum women with significant disaster exposure were at increased risk of mental health problems [34]. Xiao et al. also reported an increase in emergency department visits for mental illnesses beginning gradually after Hurricane Sandy and peaking at eight months after the storm [24].

## Geophysical events and pregnancy

Geophysical events include earthquakes and volcano eruptions. Approximately 80% of earthquakes occur at the circum-Pacific seismic belt known as the “Ring of Fire”, affecting countries such as China, Indonesia, Turkey, Iran, Japan, and the USA, as well as South American countries [35]. There were nine major earthquakes in 2020 (magnitude of greater than seven), with the most deadly quake affecting Turkey and Greece in October [36]. There are approximately one thousand five hundred potentially active volcanoes worldwide, also predominantly situated along the Ring of Fire [37]. Scientists have suggested that severe weather events such as drought and heavy precipitation may lead to increased numbers of lower magnitude or “slow” earthquakes [38, 39]. Hydraulic fracturing or “fracking”, a process used by the fossil fuel industry to extract oil and gas from underground shale formations, causes small earthquakes and may also be linked to larger earthquakes [40].

Earthquakes and volcano eruptions cause displacement, stress, and difficulty accessing health services, with volcano eruptions also causing air pollution.

### Low birth weight

Geophysical events have been linked to low birth weight in several areas around the world. Harville et al. examined the effects of several earthquakes in their systematic review [21]. A study by Tan et al. looking at perinatal outcomes after a major earthquake in Wenchuan, China, in 2008 found earthquake exposure was associated with a multitude of adverse outcomes, including a significant increase in low birth weight [41]. Higher rates of low birth weight were seen following a large earthquake in Taiwan in 1999 [21]. Oyarzo et al. found a significant increase in low birth weight after a 2010 Chilean earthquake, particularly with first trimester earthquake exposure [42]. This cause of low birth may be related to prenatal stress or exposure to environmental pollutants, but there is also evidence linking earthquakes and its impact on mental health to subsequent significantly lower neonatal birth weight [43]. No association between earthquake exposure and low birth weight or SGA was found following the Great East Japan earthquake [44, 45].

### Preterm birth

Tan et al. found that the 2008 earthquake in Wenchuan, China, was associated with a significant increase in preterm birth [41]. While Tan et al. looked at pregnancy outcomes in the year directly before and after the earthquake (2007–2008 and 2008–2009), a study by Lian et al. examined the effects of the Wenchuan earthquake on pregnancy outcomes for those exposed 6 to 8 years before conception [46]. Looking at over 70,000 births between 2015 and 2016, the authors found a significant increase in the risk of preterm birth for those with earthquake exposure compared with those not exposed. Oyarzo et al. also reported increased rates of preterm birth, as did another Chilean study by Torche et al., which looked at the sex-specific effects of exposure to natural disasters in early pregnancy [42, 47]. This study reported an increase in preterm delivery with first trimester earthquake exposure, particularly with female gender pregnancies. Increase in preterm birth was seen following a number of other earthquakes in Israel and California, with one study in Los Angeles again noting this effect was strongest for those exposed in the first trimester [21, 48]. A study in Uruguay found that exposure in the third trimester to particulate matter from volcano eruptions may be linked to preterm birth [49]. However, a study in New Zealand in 2019 failed to show an association between earthquake exposure and preterm birth [50], as did the study looking at the Great East Japan earthquake [45].

### Hypertensive disorders

Earthquake exposure was a significant risk factor for hypertensive disorders of pregnancy for women in their third trimester living in areas affected by the Great East Japan Earthquake 2011 [51].

### Psychological comorbidity

Earthquake exposure has been linked to mental health issues in pregnant women, the most commonly reported being depression and PTSD [52, 53]. A systematic review reported rates of antenatal depression amongst those who had experienced an earthquake ranging from 7 to 40%, compared to 7–13% in those who had not experienced an earthquake recently [53]. As mentioned previously, a 2015 study in Nepal found that increased exposure to earthquake led to higher rates of mental health disorders [43].

## Drought and pregnancy

The effects of drought on pregnancy are harder to examine, as drought is an insidious process without a clear beginning or end point. Droughts occur worldwide and affect an estimated fifty-five million people globally every year, with effects more severely seen in developing countries. The WHO reports that as many as seven hundred million people are at risk of being displaced by drought by 2030 [54]. Reduced access to clean water supplies and crop failure caused by drought can cause maternal dehydration and malnutrition, as well as increasing risk of exposure to water-borne illnesses. The known effects of dehydration and malnutrition on pregnancy, such as low birth weight, preterm birth, and anaemia, could be considered to be amplified in the drought setting [55, 56].

## Wildfires and pregnancy

A wildfire is an uncontrolled fire that burns in wildland vegetation. Wildfires can be started by natural causes, such as lightning or lava, or by human factors. Climate factors such as rising temperatures and drought create conditions that allow these fires to spread rapidly with devastating effects. Late 2019 and 2020 saw unprecedented bushfires spread through Australia, burning an estimated 18·6 million hectares, destroying almost three thousand homes and killing thirty-four people [57]. The 2020 wildfire season in California was the largest on record, burning 4.3 million acres and costing over twelve billion dollars in damages. The National Geographic reports that “an average of 72,400 wildfires cleared an average of 7 million acres of US land each year since 2000, double the number of acres scorched by wildfires in the 1990s” [58]. Climate change is a key factor in the increasing incidence, size, and intensity of wildfires, with wildfires breaking out in recent years in Europe, South America, and even the Arctic [59, 60]. Wildfires lead to significant air pollution, including release of particulate matter (PM), aromatic hydrocarbons, carbon monoxide, aldehydes, and other volatile organic compounds [61]. The effects of air pollution on pregnancy are well-documented in the literature—intrauterine growth restriction, low birth weight, preterm birth, and miscarriage—and so these effects may be applicable to the context of a wildfire [62–66]. Wildfires can also lead to displacement and difficulty accessing health services.

As reported by Ha, the methods used to measure exposure to wildfires include “distance to wildfires, fine particle concentration, heat spots from satellite images, and aerosol index” [12].

### Low birth weight

In the aftermath of several wildfires, the incidence of low birth weight is reported to increase. An 8-year study in Colorado found that birth weights were found to be significantly decreased after first trimester exposure to wildfire smoke [61]. A recent meta-analysis looked at eight studies evaluating wildfire exposure effect on pregnancy outcomes in four countries (USA, Australia, Indonesia, and Brazil). [67]. Six studies reported a significant link with birth weight reduction, with exposure in the second and third trimester having the greatest effect when looking at maternal proximity to wildfire-affected areas [67]. However, two studies looking at the risk of SGA found no significant relationship between wildfire exposure and SGA [61, 68]. After the 2003 bushfire in California, pregnant women exposed to wildfires had a slight reduction in birth weight compared to those without exposure [69]; however, the reduction was not statistically significant.

### Preterm birth

Wildfires have been linked to risk of preterm birth. Studies on obstetric outcomes after the above-mentioned wildfires in Colorado and Australia found links between wildfire smoke exposure preterm birth in both second trimester exposure [61] and exposure at any gestation [61, 68]. Interestingly, the effect appeared bimodal. Smoke exposure was also linked to prolonging of pregnancy past 41 weeks gestation [68]. They postulated that a perceived lack of control in the wake of a disaster may have influenced women’s decisions regarding obstetric interventions for birth. Exposure to wildfire smoke in the first or second trimester in certain regions of Brazil increased the risk of preterm birth [70]. A recent study in California looking at three million births found a significant relationship between smoke exposure and preterm birth, with the largest effects associated with second trimester exposure [71]. The authors estimated that 3.7% of preterm births in their sample were attributable to wildfire smoke exposure. Amjad et al. reported in their meta-analysis that two other studies looking at links with preterm birth did not find a significant relationship [67].

### Hypertensive disorders

Gestational hypertension was found to be linked to wildfire smoke exposure, with first trimester exposure, second trimester exposure, and exposure over the entire pregnancy showing a positive association [61].

### Gestational diabetes

Some studies have found a significant positive association between wildfire smoke and gestational diabetes with first trimester exposure [61]. Exposure to air pollution from a coal mine fire in Australia was associated with a 16% increased likelihood of gestational diabetes [72]. In a study looking at the effects of wildfires on male birth weight in Australia, O’Donnell et al. found significantly higher birth weights in males born in the most affected areas compared with less-exposed peers [73]. The authors suggest raised blood glucose levels from a maternal stress response as the cause of this finding, even in the absence of a diagnosis of gestational diabetes.

### Congenital abnormalities

Requia et al. looked at 7595 babies born in Brazil from 2001 to 2018. Their results suggest a statistically significant increased risk of cleft lip/palate, respiratory, and nervous system anomalies associated with exposure to air pollution from wildfires [74]. A study in California found an increased risk of gastroschisis with first trimester wildfire exposure [75].

## Timing of exposure

A number of studies commented on the timing of exposure to disaster in relation to adverse pregnancy outcomes. First trimester exposure to flooding was found to be most positively associated with low birth weight by one study [20]. Parayiwa et al. found a significantly increased risk of preterm birth with first trimester cyclone exposure, while second trimester exposure was linked to low birth weight [18]. A number of studies found earthquake exposure in the first trimester to be most significant for risk of preterm birth, while Balsa et al. found an increase in preterm birth following volcanic air pollution exposure in the third trimester [21, 47–49]. Third trimester earthquake exposure was a significant risk factor for hypertensive disorders of pregnancy for women affected by the Great East Japan Earthquake 2011 [51]. Requia et al. reported first or second trimester exposure to wildfire smoke was associated with risk of preterm birth [70], while second trimester exposure was found to be most significant for preterm birth by Heft-Neal et al. [71]. First trimester exposure to wildfires was again identified as the most significant timing for risk of low birth weight, gestational diabetes, and congenital anomalies [61, 75]. The study by Lian et al. in Wenchuan was notable for its examination of disaster exposure in the years before conception, with a significantly increased risk of preterm birth conferred to those with pre-conception earthquake exposure [46]. Their findings indicate a need for further study of the long-term reproductive effects of disaster exposure.

## Potential pathophysiology

The adverse effect of environmental factors on many of these outcomes is generally thought in the literature to be linked to increased prenatal stress. This can be related to the direct hormonal effect of stress, through increased release of corticotropin-releasing hormone, cortisol, and inflammatory cytokines. It is also important to consider the indirect effects of prenatal stress—impaired immune system response, increased risk of infection, and increased rates of smoking seen in the wake of disasters—as potential aetiology for these outcomes. Exposure to environmental toxicants may be implicated in developing perinatal complications, as seen with seawater in Bangladesh [26]. Other toxicants such as toxic chemical waste, household moulds, and carbon monoxide are often released in the wake of natural disasters and may have pathological effects.

## Discussion

With climate change continuing to accelerate and environmental migration rising, most maternity healthcare providers are likely to provide care for domestic or international environmental migrants over the coming years. More than ninety million international migrants are residing in the World Health Organization (WHO) European region and more than half of these migrants are women, many of childbearing age [5]. Many of these may have environmental reasons behind their migration.

The WHO recognises a lack of global or region-wide indicators for refugee and migrant health and a lack of framework for routine collection of data, pointing out that “this leads to a shortage of scientifically valid and comparable health data on refugee and migrant populations” [76]. There is no existing data on pregnant environmental migrants, or indeed on environmental migrants in general. Most of the studies examined in this article looked at women staying in the disaster-affected region. As such, we can only postulate on the perinatal risks of environmental migration, by extrapolating the existing evidence on the effects on pregnancy of (a) migration and (b) natural disasters.

### Perinatal health effects

Environmental disasters have complex effects on perinatal health. The most consistently reported effects are low birth weight and mental health issues. An association with preterm birth is frequently suggested by observational studies, but results are inconsistent. It is important to consider these findings in the context of the existing increased risk of mental health issues and preterm birth faced by pregnant migrant women [6]. A synergistic effect may occur. Gestational diabetes and hypertensive disorders are other important complications to consider, along with maternal malnutrition.

The social factors of displacement and migration add a layer of complexity to the care of these patients. In 2018, the WHO released a policy brief entitled “Improving the health of pregnant refugee and migrant women and newborn children” [76]. This policy outlines that socioeconomic challenges faced by pregnant refugees and migrant women are the greatest determinant of mother and infant health. These women are often living in poor conditions or find themselves facing financial difficulties. A systematic review by Fair et al. conducted in Europe on migrant women’s experience of pregnancy reported that many migrant women had difficulty covering costs of basic living, transport to antenatal visits, and costs of essential care [77]. Accessibility to healthcare is a major challenge, with language, a lack of trust in healthcare providers, and unfamiliar healthcare practices reported as some of the key barriers for migrant women.

### A checklist for care

**Fig. 1:**
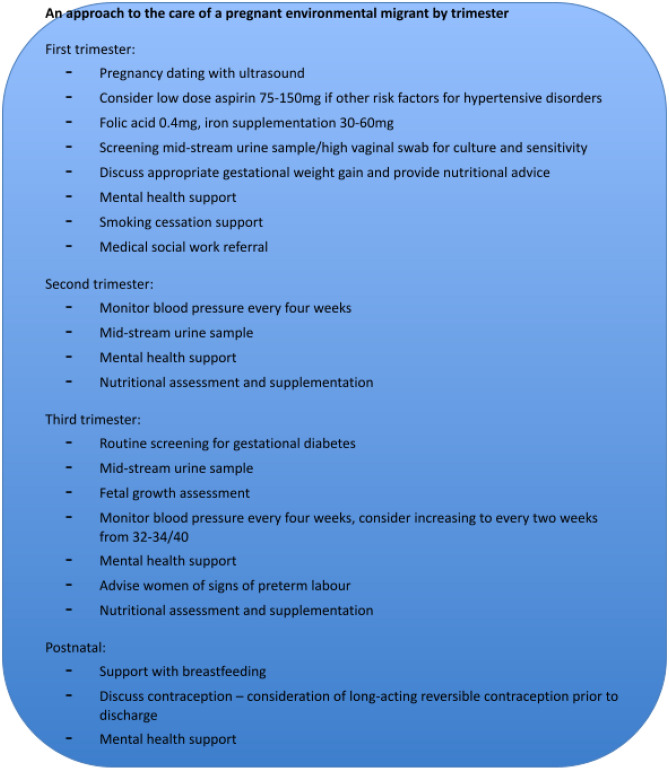
A proposed checklist for care

Based on the findings suggested by this review and the relevant social factors described above, we have developed a proposed checklist for care to use in the assessment of a pregnant environmental migrant (Fig. 1).

All patients should have a first trimester ultrasound dating scan. Given the potential increased risk of hypertensive disorders with disaster exposure, we suggest consideration of the use of low-dose aspirin if other significant risk factors for hypertensive disorders are present. The migration process is associated with poor living conditions and exposure to suboptimal hygiene facilities. It is important that any woman with a history of recent migration has a nutritional assessment and is prescribed folic acid and iron supplementation. A screening mid-stream urine (MSU) sample and high vaginal swab should be sent for culture and sensitivity. As discussed, these patients are at increased risk of mental health issues; referral to perinatal mental health services should be offered, alongside smoking cessation support. Social work referral should be considered.

In the second trimester, mental health support and nutritional supplementation should continue. Blood pressure should be monitored monthly. MSU sample should be sent for culture and sensitivity.

In the third trimester, we suggest routine screening for gestational diabetes. The risk of low birth weight in this population means consideration should be given to a fetal growth scan at 32–34 weeks. Blood pressure should be monitored four-weekly up until 32–34 weeks, at which stage fortnightly monitoring is encouraged. The potential risk of preterm birth means signs of preterm labour should be discussed and advice provided. Mental health support and nutritional supplementation should continue. MSU sample should be sent.

In the postnatal period, support with breast-feeding should be provided. Patients should be offered contraceptive advice, with consideration of a long-acting reversible contraceptive prior to discharge. A final mental health assessment should be carried out, with arrangements for ongoing support made for those who require it. Discharge planning should take into consideration the social supports available to or required by the woman.

Depending on how recently or how far these patients have migrated, they may not have a general practitioner in the community. It is important to ensure continuity of care and regular attendance at antenatal check-ups across the three trimesters, as well as postnatal support.

### Migration as a protective factor?

However, we must also consider the potential protective role of migration. For example, women who migrate early from regions affected by air pollution or flooding may reduce their risk of adverse perinatal outcomes. As mentioned above, prenatal and first trimester exposure to disasters are significant for conferrance of risk. Timing of migration will be a critical factor in determining perinatal outcomes. It may be important to develop a twofold approach to the care of environmental migrants—a short-term approach to those who have recently migrated or migrated during the course of their pregnancy, and a longer-term approach to those who migrated in time to mitigate their risk. Further research is required to assess the time period necessary to provide a protective effect.

### Improving migrant care and opportunities for future research

The 2018 WHO policy briefly recognises the increased perinatal morbidity and mortality for pregnant migrants, and states that these women tend to experience suboptimal perinatal care [76]. It provides a number of recommendations to improve care for these women, including increasing healthcare professionals’ awareness of disease burden in specific migrant groups and how these can affect pregnancy outcomes, and including screening where indicated. Fair et al. also called for research on the needs of different migrant populations to develop tailored interventions [77]. Our paper, by considering a specific migrant group and studying the disease burden faced by this group, will improve the awareness of obstetricians worldwide and provide tailored screening advice for these women. It is important to acknowledge that additional funding may be required to implement the necessary care pathways for increasing numbers of environmental migrants worldwide.

Going forward, this paper has flagged a number of potential research opportunities. Focused research on environmental migrants and specifically pregnant environmental migrants is required. Research on the relationship between perinatal complications and timing of migration is likely to be of particular importance. Future studies on the care of pregnant migrants may wish to use this paper to consider environmental migrants as an important subgroup.

## Conclusion

Environmental migrants may have specific risks of perinatal adverse outcomes in pregnancy. Maternity healthcare providers should familiarise themselves with the potential impact of environmental migration on pregnancy and the specific perinatal risks faced by the pregnant environmental migrant. Recognising the needs of these patients will allow obstetricians and midwives to provide individualised care and offer the additional supports required.

Furthermore, the WHO has identified climate change as “the single greatest health threat facing humanity” [78]. The 2015 Paris Agreement recognised that women are disproportionately affected by climate change [79]—as such, it follows that women’s health globally is disproportionately affected. A recent article by the International Federation of Gynaecology and Obstetrics (FIGO) recognises the climate crisis as a risk to women, pregnant mothers, and their fetuses, and outlines the role maternity healthcare providers must play in climate advocacy, research, education, and patient care [80]. It is imperative that obstetricians, midwives, and maternity healthcare providers worldwide use their platform to highlight the effects of climate change on the pregnant population and to advocate for global action to address climate change, for the safety of patients and future populations.
